# Multiple Aflatoxins Drive Cumulative Dietary Exposure and Hepatocellular Carcinoma Risk: An Age-Stratified Study in Guangzhou, China

**DOI:** 10.3390/foods15111839

**Published:** 2026-05-22

**Authors:** Qian Huang, Yanyan Wang, Yan Li, Yixuan Xu, Yuhua Zhang, Lan Liu, Jinheng Zeng, Weiwei Zhang, Yan Yang

**Affiliations:** 1 Department of Nutrition, School of Public Health (Shenzhen), Shenzhen Campus of Sun Yat-sen University, Shenzhen 518107, China; huangq285@mail2.sysu.edu.cn (Q.H.); xuyx63@mail2.sysu.edu.cn (Y.X.); 2 Department of Food Safety and Nutrition, Guangzhou Center for Disease Control and Prevention (Guangzhou Health Supervision Institute), Guangzhou 510440, China; wangyy13213845@163.com (Y.W.); gzcdcliy@foxmail.com (Y.L.); pisceszyh@126.com (Y.Z.); liulan728@163.com (L.L.); zjh578508814@163.com (J.Z.); 3 Department of Environmental Sanitation, Guangzhou Center for Disease Control and Prevention (Guangzhou Health Supervision Institute), Guangzhou 510440, China; 4 Guangdong Engineering Technology Research Center of Nutrition Transformation, Sun Yat-sen University, Shenzhen 518107, China; 5 Guangdong Provincial Key Laboratory of Food, Nutrition and Health, Sun Yat-sen University, Guangzhou 510080, China

**Keywords:** aflatoxins, exposure assessment, hepatocellular carcinoma, food safety

## Abstract

Aflatoxins are widespread hepatotoxic food contaminants, yet age-specific cumulative exposure to multiple aflatoxins and associated health risks remain poorly characterized. This study assessed cumulative dietary exposure to aflatoxin B_1_ (AFB_1_), B_2_, G_1_, and G_2_, and hepatocellular carcinoma (HCC) risk across five age groups, evaluating the influence of packaging and retail sources on contamination. Contamination data of 1179 food samples and consumption data were integrated to calculate the margin of exposure (MoE) and annual HCC incidence. AFB_1_ was most frequently detected and often co-occurred with other aflatoxins; bulk vegetable oils showed the highest total aflatoxin detection rate. Roasted peanuts contributed most to aflatoxin exposure, particularly among children aged 3–6 (MoE 900–1206). Rice, rice products, and coarse grains were primary contributors to aflatoxin-attributable HCC risk (0.008 cases per 100,000 person-years). Overall contamination was significantly higher in bulk products than in pre-packaged foods (*p* < 0.05) and in samples from farmers’ markets and grocery stores than in other sites (*p* < 0.05). These findings reveal non-negligible aflatoxin-related health risks for Guangzhou residents, especially young children and frequent consumers of staple grains and nuts. Targeted monitoring of high-risk foods and retail environments and age-specific dietary guidance are recommended to reduce population-level aflatoxin exposure and HCC risk.

## 1. Introduction

Food safety remains a critical public health concern worldwide, with mycotoxin contamination representing a persistent challenge to the safety of agricultural products. Among contamination sources, aflatoxins are of particular concern due to their high toxicity, widespread occurrence, and strong carcinogenic potential. Globally, aflatoxin exposure imposes a substantial health burden: the World Health Organization (WHO) estimates that approximately 55,000 annual deaths from hepatocellular carcinoma (HCC) are directly related to aflatoxin exposure, primarily in developing countries [1]. Economically, aflatoxin contamination imposes a substantial burden on food systems, with global annual losses estimated at USD 6–18 billion due to healthcare costs and lost productivity [2].

Aflatoxins are secondary metabolites primarily produced by *Aspergillus flavus* and *Aspergillus parasiticus*, classified as Group 1 human carcinogens by the International Agency for Research on Cancer [3]. They frequently contaminate staple foods such as maize, peanuts, and rice, especially when crops are cultivated, stored, or transported under warm and humid conditions. Chronic dietary exposure to aflatoxins has been associated with hepatotoxicity, immunosuppression, growth retardation, and most notably, hepatocellular carcinoma.

Among more than 20 identified aflatoxins, aflatoxin B_1_ (AFB_1_) is the most toxic and carcinogenic, primarily promoting HCC following hepatic metabolism [4]. In addition to AFB_1_, aflatoxin B_2_ (AFB_2_), aflatoxin G_1_ (AFG_1_), and aflatoxin G_2_ (AFG_2_) are also frequently detected in cereals and nuts, while aflatoxin M_1_ (AFM_1_) is mainly found in dairy products. Most previous risk assessments have focused on single aflatoxin exposure, particularly AFB_1_, due to its high carcinogenic potency. However, AFB_1_, AFB_2_, AFG_1_, and AFG_2_ often co-occur in food matrices, with AFB_1_ and AFB_2_ being the most commonly reported co-contaminants [5], leading to multi-aflatoxin co-exposure. Although AFB_2_, AFG_1_, and AFG_2_ individually exhibit lower toxicity, their co-occurrence with AFB_1_ may result in additive or synergistic effects. Gao et al. have reported that AFM_1_ can exacerbate AFB_1_-induced intestinal barrier damage, highlighting the public health relevance of multi-aflatoxin exposure [6]. Dietary aflatoxin exposure often coexists with chronic hepatitis B virus (HBV) infection. These two factors are identified as the two dominant drivers of elevated HCC incidence observed in low- and middle-income countries [7]. In China, this dual burden is particularly evident in Guangdong Province, where 94.46% of hepatitis B cases were chronic infections [8], emphasizing the need for assessing aflatoxin-related HCC risk. Moreover, lifespan differences in dietary preferences and physiological vulnerability necessitate a stratified risk assessment.

Aflatoxin contamination is widely distributed across the globe, with a particularly high prevalence in Africa and Asia, where climatic conditions and post-harvest practices favor fungal growth [9]. In China, aflatoxin contamination is most pronounced in eastern, southern, and central regions, where peanuts, peanut oil, and cereal products are primarily contaminated [10]. Aflatoxin-related trade restrictions have further highlighted the significance of food safety governance: over five years, peanut product exports exceeded EU legal limits on more than 180 occasions [11], demonstrating that effective mycotoxin control is crucial not only for protecting public health but also for ensuring compliance with international trade standards.

In Guangzhou, a humid coastal megacity in southern China, high annual temperatures and precipitation [12] create favorable conditions for *Aspergillus* growth, providing a representative setting for dietary aflatoxin exposure assessment. As a major grain import hub, Guangzhou relies on imported cereals and oilseeds, which introduces contamination risks during long-distance storage. In addition, increased consumption of nuts and edible oils has diversified exposure pathways, potentially increasing population-level aflatoxin intake. Although China has established regulatory limits for aflatoxins, non-compliant samples are still detected. Peanuts and peanut oil consistently exhibit the highest detection rates nationwide [13]. A recent survey in Guangdong province revealed bulk edible oils collected from residents’ kitchens had AFB_1_ detection and exceedance rates of 79.72% and 60%, respectively [14]. Beyond climatic influences, packaging type (bulk versus pre-packaged) and retail source are increasingly recognized as critical yet insufficiently studied determinants of aflatoxin contamination.

Despite extensive research on aflatoxin contamination, several critical gaps remain in Guangzhou’s exposure scenario. Most existing risk assessments focus exclusively on AFB_1_, neglecting the frequent co-occurrence of multiple aflatoxins and their potential combined toxicity. Moreover, age-specific exposure and health risk assessments are limited, resulting in insufficient protection for vulnerable populations such as children and older adults.

To address these gaps, this study integrates multi-aflatoxin food surveillance data (2016–2023) with dietary consumption patterns in Guangzhou to assess (1) age-specific cumulative dietary exposure and health risks using the margin of exposure (MoE) approach and HCC risk models; (2) identify high-risk foods and vulnerable populations; and (3) evaluate the influence of packaging types and retail sources on contamination levels. In this study, single-aflatoxin exposure is defined as exposure to each of the four aflatoxins (AFB_1_, AFB_2_, AFG_1_, and AFG_2_) individually, whereas multi-aflatoxin co-exposure refers to simultaneous exposure to all four aflatoxins. The findings are expected to provide scientific evidence for risk-based food safety regulation and targeted public health interventions.

## 2. Materials and Methods

### 2.1. Study Design and Data Sources

This study is a retrospective dietary exposure assessment based on food surveillance and consumption data. Aflatoxin monitoring data were obtained from the Guangzhou Food Safety Risk Monitoring Program from 2016 to 2023. Since detailed dietary consumption patterns for the corresponding population were not available within the same program, the most recent comprehensive local dietary survey data were utilized for exposure calculation, based on the assumption that core dietary structures for staple foods remain relatively stable over time [15], as detailed in Section 2.4.

### 2.2. Sample Collection

From 2016 to 2023, a total of 3117 food samples were randomly collected following the sampling framework of the China National Food Contamination and Harmful Factors Risk Monitoring Program from multiple areas in Guangzhou. The samples covered six major categories susceptible to mycotoxin contamination: rice and rice products, flour and flour products, starch foods, coarse grains, vegetable oils, and nuts. For cumulative exposure assessment, 1179 samples were purposively selected from high-risk categories under the same randomized surveillance framework and analyzed for all four aflatoxins: AFB_1_, AFB_2_, AFG_1_, and AFG_2_, while the remaining samples were allocated to other monitoring objectives. To compare the differences in aflatoxin levels between bulk and pre-packaged forms of rice/rice products versus flour/flour products and to calculate the HCC risk from dietary exposure to AFB_1_ alone, we analyzed data from 2763 samples with measured AFB_1_ concentrations. The specific sub-categories and sample sizes included bulk vegetable oil (*n* = 697), pre-packaged vegetable oil (*n* = 40), roasted peanuts (*n* = 139), other roasted nuts (*n* = 207), raw peanuts (*n* = 46), and other raw nuts (*n* = 50). Other nuts include walnuts, sunflower seeds, and similar products.

Samples were collected by professionally trained researchers simulating consumer purchase behaviors from major food retail sources, including supermarkets, farmers’ markets, grocery stores, food production workshops, and online stores. The sampling strategy ensured spatial representation across both urban and rural districts of Guangzhou, with representation from all major brands within each food category.

### 2.3. Sample Preparation and Chemical Analysis

Individual stock solutions (1 μg/mL) of the four aflatoxins were prepared in acetonitrile. Each stock solution, along with isotope-labeled internal standards, was mixed and diluted with acetonitrile to prepare intermediate standards (0.5 µg/mL) and a working solution (0.01 µg/mL). All solutions were stored in darkness at −20 °C until analysis.

Samples exceeding 500 g were collected, ground, sieved, and quartered to obtain 100 g subsamples for sealed storage. Then, 5.00 g of the sample was accurately weighed into a 50 mL centrifuge tube, followed by the addition of 20 mL of acetonitrile–water–formic acid (70:29:1, *v*/*v*/*v*). The mixture was vortexed for 1 min and then shaken on an orbital shaker for 30 min. Subsequently, 1.0 mL of the extract was transferred to a 1.5 mL tube and centrifuged at 10,000 rpm for 5 min. An aliquot of 0.5 mL of the supernatant was transferred to another 1.5 mL tube and mixed with 1.0 mL of water. After vortex mixing, the solution was centrifuged at 10,000 rpm and 4 °C for 5 min. The resulting supernatant was filtered through a 0.22 μm membrane. Finally, 180 μL of the filtrate was transferred to a 300 μL insert, mixed with 20 μL of the mixed isotope-labeled internal standard solution, vortexed, and subjected to instrumental analysis.

Aflatoxins in each food sample were quantified using isotope dilution liquid chromatography–tandem mass spectrometry (ID-LC-MS/MS), as per the Manual for China National Food Contamination and Harmful Factors Risk Monitoring. Measurements were performed using an ultra-high-performance liquid chromatography–tandem mass spectrometer equipped with an electrospray ionization source. Chromatographic separation of mycotoxins was carried out on a Waters BEH C18 column (column length 150 mm × 2.1 mm; 1.7 µm particle size), or an equivalent column, maintained at 40 °C with a flow rate of 0.3 mL/min. The mobile phases were (A) 0.2% formic acid in water and (B) acetonitrile. The gradient elution program was as follows: 0 min, 20% B; 1 min, 20% B; 4 min, 40% B; 10 min, 70% B; 10.2 min, 100% B; 11.8 min, 100% B; 12 min, 20% B; 15 min, 20% B. The injection volume was 10 μL.

Each sample was analyzed in duplicate. The absolute difference between the two results under repeatability conditions should not exceed 23%. Spike recovery tests were performed during detection. The limits of detection (LOD) for AFB_1_, AFB_2_, AFG_1_, and AFG_2_ were 0.1, 0.1, 0.2, and 1 μg/kg, respectively, as per the China National Food Safety Standard for Maximum Levels of Mycotoxins in Foods (GB 2761-2017) [16], with limits of quantitation set at three times the LOD. Spike recoveries were required to fall within the range of 60–120%.

### 2.4. Food Consumption Data

Dietary consumption data were derived from the results of a 2011 survey on trans fatty acid intake among residents in the Guangzhou area [17,18]. Following the requirements of the China National Chronic Disease and Nutrition Surveillance Work Plan, a multi-stage stratified random sampling method was used to select 1000 households (approximately 3000 people) from 7 administrative districts in Guangzhou for a 3-day 24 h dietary recall to obtain consumption data for various food categories. The population was categorized into five age groups: young children (3–6 years), children and adolescents (7–17 years), adults (18–59 years), the elderly (≥60 years), and the whole population. To control for potential recall bias inherent to the 24 h dietary recall method, all interviewers received standardized training on probing techniques and portion size estimation. Face-to-face interviews were conducted by trained dietitians. Portion size estimation was assisted using food models and standardized utensils. Dietary recalls were collected over three consecutive days. For children aged <7 years or individuals with limited recall ability, a primary caregiver served as a proxy respondent to provide dietary recall information. After the open-ended recall, a pre-specified food checklist was used to verify and supplement commonly omitted food items.

Guangzhou’s population maintains a region-specific diet deeply rooted in Lingnan culture. According to the Guangzhou Bureau of Statistics, food consumption among urban and rural residents showed negligible fluctuation between 2012 and 2023 [19]. Thus, data collected in 2011 can reasonably represent dietary intake for the subsequent period spanning 2016 to 2023.

### 2.5. The Maximum Limit Levels for Aflatoxins in Different Food Groups

To evaluate compliance, results were compared against the China National Standard (GB 2761-2017) [16] and Codex Alimentarius (Codex Standard 193-1995) [20]. Total aflatoxins (AFT) in this study refer to the sum of AFB_1_, AFB_2_, AFG_1_, and AFG_2_. The regulatory standard details are shown in Table 1. Treatment of left-censored data (values below LOD) followed the European Food Safety Authority (EFSA) recommendations for managing analytical non-detects (ND) [21]: “upper bound” (UB, ND = LOD), “middle bound” (MB, ND = 1/2 LOD), and “lower bound” (LB, ND = 0) scenarios were performed to express the interval of exposure.

### 2.6. Dietary Exposure Assessment

The dietary exposure to AFT was calculated using the estimated daily intake (EDI) as the indicator. The calculation formula is as follows:

(1) $$\mathrm{EDI}\;=\;\sum \left(D_{i}\;\times \;C_{i}\right)/W\;$$

EDI is the estimated daily intake of AFT for each age group (ng/kg·bw); *D_i_* is the concentration of AFT in food category i (μg/kg), *C_i_* is the daily consumption of food category i (g/day), and *W* is the average body weight of each age group (kg). Due to differences in dietary aflatoxin exposure among age groups [2], we analyzed the whole population and different age groups separately. The average body weights for each age group were as follows: 20 kg for populations aged 3–6 years, 40 kg for those aged 7–17 years, 62 kg for those aged 18–59 years, and 60 kg for those aged ≥60 years and the whole population [22,23,24]. Since AFB_2_, AFG_1_, and AFG_2_ exhibit structural and toxicity-mechanistic similarities to AFB_1_, the EDI for AFT was the sum of EDIs of the four aflatoxins [25].

### 2.7. Health Risk Assessment

Since aflatoxins are carcinogenic and have no available health-based guidance values for comparison, the MoE method was used to assess the cumulative dietary exposure risk of the four aflatoxins: AFB_1_, AFB_2_, AFG_1_, and AFG_2_. The MoE was calculated as follows:

(2) $$MoE={BMDL}_{10}/EDI$$

The Benchmark Dose Lower Confidence Limit for a 10% Response (BMDL_10_) is the lower limit of the 95% confidence interval for the dose causing tumors in 10% of animals, based on dose–response relationship results from animal studies. A BMDL_10_ of 400 ng/kg·bw was used for AFB_1_, according to the CONTAM Panel scientific opinion, but for AFB_2_, AFG_1_, and AFG_2_, due to limited data in vivo to derive their carcinogenic potency factors, they are generally assumed to have the same carcinogenic potency as AFB_1_ [26]. This conservative assumption may overestimate the actual cumulative risk, as other aflatoxins are generally considered less potent carcinogens than AFB_1_. Therefore, the cumulative risk estimates presented here should be interpreted as upper-bound, conservative estimates. An MoE < 10,000 indicates a potential public health concern. For cumulative risk, the combined MoE (MoE_total_) was derived from the potency-weighted sum of individual aflatoxins, assuming equal carcinogenic potency to AFB_1_ as a conservative approach:

(3) $${\mathrm{MoE}}_{\mathrm{total}}=1/(1/{\mathrm{MoE}}_{{\mathrm{AFB}}_{1}}\;+\;1/{\mathrm{MoE}}_{{\mathrm{AFB}}_{2}}\;+\;1/{\mathrm{MoE}}_{{\mathrm{AFG}}_{1}}\;+\;1/{\mathrm{MoE}}_{{\mathrm{AFG}}_{2}})$$

### 2.8. Hepatocellular Carcinoma Risk Assessment

Referring to the formula proposed by the Food and Agriculture Organization of the United Nations and the WHO [17], the hepatocellular carcinoma risk induced by AFB_1_ and AFT exposure was quantitatively estimated. The formula combines the carcinogenic potency for hepatitis B surface antigen-positive (HBsAg^+^) individuals and negative (HBsAg^−^) individuals with EDI of AFB_1_ (ng/kg). The carcinogenic potency is expressed as the number of HCC cases per 100,000 individuals annually per ng/kg·bw of daily AFB_1_ exposure, with values of 0.3 and 0.01 for HBsAg^+^ and HBsAg^−^ individuals, respectively [27]. The percentage of HBsAg^+^ individuals in the population aged 1–59 years in Guangzhou in 2018 was 9.50% [28]. Based on the following formula, the hepatocellular carcinoma risk for each age group was calculated as follows:

(4) $$P_{\mathrm{HBsAg}+}\;=\;\mathrm{EDI}\;\times \;0.3$$

(5) $$P_{\mathrm{HBsAg}-}=\mathrm{EDI}\;\times \;0.01\;$$

(6) $$P_{\mathrm{combined}}=\mathrm{EDI}\;\times \;[0.3\;\times \;9.50\%+0.01\;\times \;(1\;-\;9.50\%)]$$

P_HBsAg+_: HCC risk for HBsAg^+^ individuals exposed to AFB_1_ in the population; P_HBsAg−_: HCC risk for HBsAg^−^ individuals exposed to AFB_1_ in the population; P_combined_: Total HCC risk for the population exposed to AFB_1_. Based on the 9.50% HBsAg^+^ prevalence, if 1 ng AFB_1_ per kg body weight is consumed daily, this would lead to an additional 0.038 hepatocellular carcinoma cases per 100,000 people per year. As AFB_1_ is the most predominant and toxic aflatoxin, the CONTAM Panel adopted a conservative approach by equating the carcinogenic potency of AFT with that of AFB_1_ [29]. Therefore, we used the same formulas to calculate the P_HBsAg+_, P_HBsAg−_, and P_combined_ of AFT.

### 2.9. Statistical Analysis

Data were collated and analyzed using Excel 2021 and R 4.4.1. The median, along with the 25th and 75th percentiles (P25, P75), was used to describe the average aflatoxin concentration. Food consumption data are presented as mean ± standard deviation (SD). The Mann–Whitney U test was used to compare whether aflatoxin levels differed between foods by different packaging types. The Kruskal–Wallis H test was used to compare foods collected from different retail sources. Post hoc multiple comparisons were performed using the Dunn test. *p*-value < 0.05 was considered statistically significant.

## 3. Results

### 3.1. Occurrence and Contamination Levels of Aflatoxins in Food Groups

**Figure 1:**
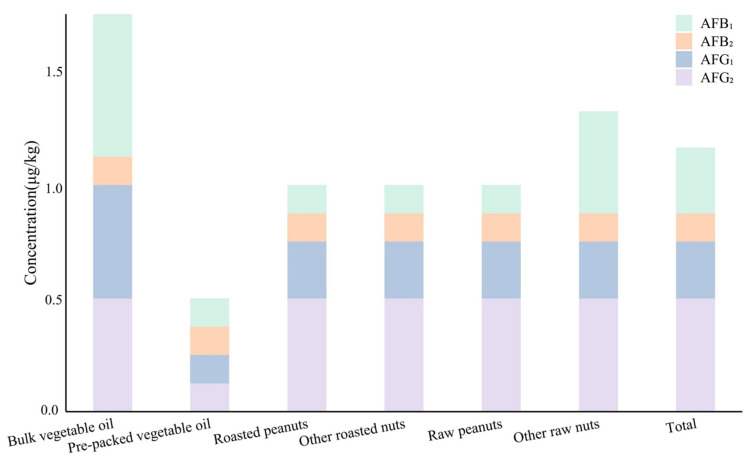
The detected concentration and distribution of each aflatoxin among various and total food categories. The cumulative levels of the four aflatoxins in each food category are shown as a stacked bar chart, with each color representing the median of the respective toxin based on middle bound values.

Among the 1179 food samples analyzed for aflatoxins, AFG_2_ exhibited the highest median contamination level across all samples. When comparing food categories, bulk vegetable oil showed the highest cumulative aflatoxin concentrations, with AFB_1_ being the predominant aflatoxin, whereas pre-packaged vegetable oil exhibited the lowest concentration levels (Figure 1).

The detection rates and median contamination levels of the four individual aflatoxins and AFT, as well as the exceedance rates of AFB_1_, are summarized in Supplementary Table S1. Notably, all four aflatoxins were found to co-occur in 52.84% (623/1179) of the samples. Specifically, AFB_1_ was the most frequently detected aflatoxin, with a detection rate of 51.74% (610/1179), followed by AFB_2_ at 25.45% (300/1179). By contrast, AFG_1_ was rarely detected, with a detection rate of only 0.85% (10/1179).

Marked differences in aflatoxin occurrence were observed among food categories. AFT was most frequently detected in bulk vegetable oil (68.72%, 479/697), whereas the lowest detection rate was observed in raw peanuts (13.04%, 6/46). AFB_1_ was commonly detected in bulk vegetable oil (67.58%, 471/697) and other raw nuts (60.00%, 30/50). AFB_2_ also showed a relatively high detection rate in bulk vegetable oil (39.45%, 275/697). In contrast, AFG_2_ was mainly detected in pre-packaged vegetable oil (15.00%, 6/40), while AFG_1_ was detected in only a few samples.

Across all food samples, 54 samples (4.58%) exceeded the maximum limits for AFB_1_. Bulk vegetable oil exhibited a particularly high exceedance rate, with 50 samples (7.17%) exceeding the regulatory limit.

In terms of contamination levels, the highest median concentrations of AFB_1_ (0.63 μg/kg) and AFG_1_ (range: 0.00–1.00 μg/kg) were observed in bulk vegetable oil. Pre-packaged vegetable oil showed the lowest AFG_2_ concentration (range: 0.00–0.25 μg/kg). The median concentration of AFB_2_ was generally comparable across the different food groups.

### 3.2. Food Consumption of Guangzhou Residents

The consumption of major food categories susceptible to aflatoxin contamination among different age groups in Guangzhou, based on a 2011 dietary survey [14], is presented in Table 2. For the whole population, rice and rice products were the most frequently consumed foods (135.84 ± 86.94 g/day), followed by flour and flour products (49.93 ± 36.29 g/day). Peanuts showed the lowest average consumption (0.66 g/day). Age-specific differences in consumption patterns were observed. Children aged 3–6 years had a relatively higher intake of peanuts compared with other age groups, whereas individuals aged ≥60 years consumed smaller amounts of starchy foods.

### 3.3. Comparison of Aflatoxin Concentrations by Packaging Types and Retail Sources

**Figure 2:**
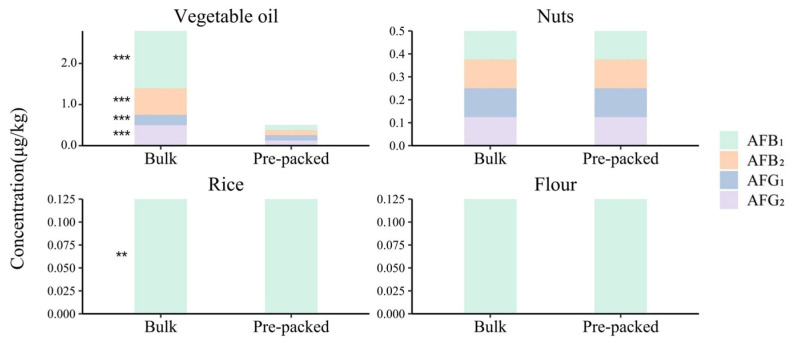
Comparison of the concentrations of four aflatoxins between bulk and pre-packaged foods. Vegetable oils and nuts were tested for AFB_1_, AFB_2_, AFG_1_ and AFG_2_, whereas rice and flour were tested only for AFB_1_. The height of each color represents the median of the respective toxin based on middle bound values. A Mann-Whitney U test was used for analysis. For aflatoxin levels between bulk and pre-packaged forms of the same food, **: *p* < 0.01; ***: *p* < 0.001.

To assess the potential influence of food characteristics on aflatoxin contamination, aflatoxin concentrations were compared between foods with different packaging types and from different retail sources. Significant differences were observed between bulk and pre-packaged vegetable oils for all four aflatoxins (*p* < 0.001), with consistently higher concentrations in bulk products. In addition, AFB_1_ concentrations in bulk rice were significantly higher than those in pre-packaged rice (*p* = 0.003) (Supplementary Table S2). No significant differences were observed in aflatoxin concentrations between bulk and pre-packaged nuts and flour products (Figure 2).

**Figure 3:**
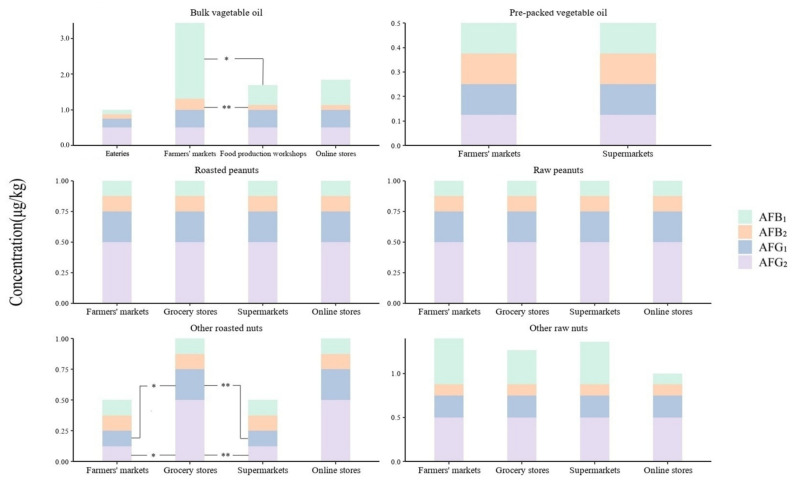
Comparison of the concentrations of four aflatoxins among foods collected from various retail sources. The height of each color represents the median of the respective toxin based on midlle bound values. Kruskal-Wallis H test and Dun’s multiple comparison test were used for analysis. For aflatoxin levels between two retail sources of the same food, *: *p* < 0.05; **: *p* < 0.01.

Differences in aflatoxin concentrations among foods collected from different retail sources, including eateries, farmers’ markets, grocery stores, supermarkets, food production workshops, and online stores, are shown in Figure 3. Multiple comparison analyses indicated that AFB_1_ (*p* < 0.05) and AFB_2_ (*p* < 0.01) concentrations in bulk vegetable oil obtained from farmers’ markets were significantly higher than those from food production workshops. In other roasted nuts, concentrations of AFG_1_ and AFG_2_ in samples collected from grocery stores were higher than those from both farmers’ markets (*p* < 0.05) and supermarkets (*p* < 0.01). Detailed statistical results are provided in Supplementary Table S3.

### 3.4. Dietary Intake of Aflatoxins and Health Risk Assessment

**Figure 4:**
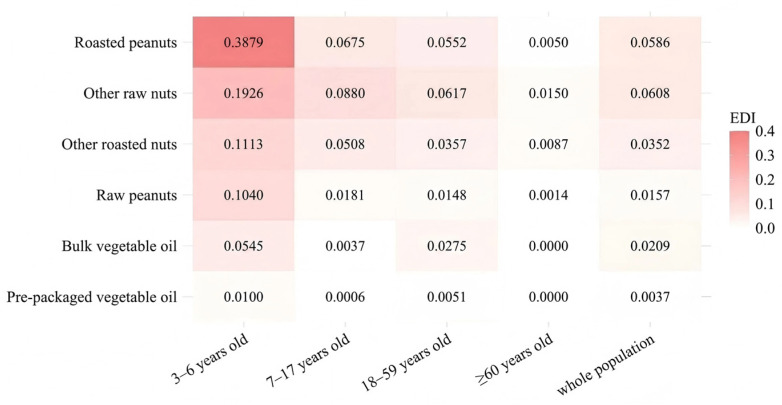
The estimated daily intake (EDl) of AFT among age groups. Increasing values and color intensity correspond to elevated aflatoxin exposure. EDI was calculated based on the middle bound values of AFT concentration.

EDIs of aflatoxins by age group, based on food consumption data and average aflatoxin concentrations, are shown in Figure 4. For the whole population, roasted peanuts and other raw nuts were the main contributors to dietary exposure to aflatoxins, with EDI values ranging from 0.0259 to 0.0956 ng/kg·bw (Supplementary Table S4). In contrast, pre-packaged vegetable oil contributed the least to total exposure (0.0028–0.0174 ng/kg·bw).

Among children aged 3–6 years, roasted peanuts represented the dominant source of dietary aflatoxin exposure, with EDI values ranging from 0.3316 to 0.4442 ng/kg·bw. For individuals aged ≥60 years, raw nuts were the main contributors to aflatoxin exposure (0.0063–0.0236 ng/kg·bw).

**Figure 5:**
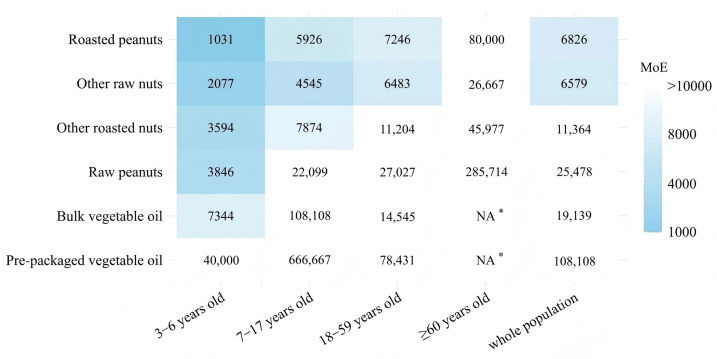
The margin of exposure (MoE) of AFT among age groups. Decreasing values and color intensity correspond to elevated risk of aflatoxin exposure. Values greater than 10,000 indicate negligible risk of aflatoxin exposure, NA * denotes MoE values > 10^6^.

Exposure risk characterization based on the MoE_total_ is presented in Figure 5. The BMDL_10_ established by EFSA for AFB_1_ was applied to all four aflatoxins due to their structural and toxicological similarities. The MoE_total_ for AFT was calculated from the individual MoEs of AFB_1_, AFB_2_, AFG_1_, and AFG_2_.

For the whole population, the MoE values for cumulative aflatoxin exposure from most food categories were well above 10,000, indicating a low level of concern. However, relatively lower MoE values were observed for roasted peanuts (5961–8002) and other raw nuts (4184–15,455). Elderly individuals showed generally high MoE values, reflecting lower exposure risks associated with reduced consumption of nuts and vegetable oils. In contrast, children aged 3–6 years exhibited comparatively lower MoE values for bulk vegetable oil, roasted nuts, and raw nuts, with the lowest MoE observed for roasted peanuts (900–1206). In addition, the exposure risk to aflatoxins is higher for roasted peanuts compared to raw peanuts, while for other nuts, such as walnuts, raw forms pose a greater exposure risk than roasted counterparts.

**Figure 6:**
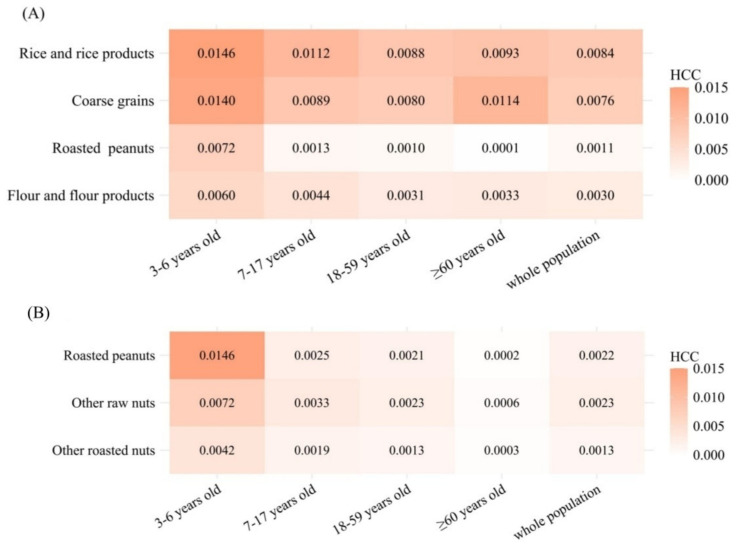
Hepatocellular carcinoma (HCC) risk associated with dietary intake of AFB_1_ (**A**) and AFT (**B**) across different age groups. Only high-risk food categories with larger MoE values for AFB_1_ and AFT are presented. Increasing values and color intensity correspond to elevated HCC risk.

Based on the MoE results for AFB_1_ (Supplementary Table S5) and AFT, food groups presenting relatively higher exposure risks were selected for HCC risk assessment (Figure 6). For the whole population, rice and rice products, as well as coarse grains, were the primary contributors to AFB_1_-induced HCC risk, each estimated to increase the incidence by approximately 0.008 cases per 100,000 person-years. Among individuals aged ≥60 years, coarse grains represented the main contributor to HCC risk, with an estimated increase of 0.011 cases per 100,000 person-years. Regarding AFT-induced HCC risk, roasted peanuts and children aged 3–6 years remained the highest-risk food and population group of concern, with an estimated lifetime additional HCC risk of 0.015 cases per 100,000 person-years as a result of early-life dietary exposure.

## 4. Discussion

This study comprehensively evaluated cumulative dietary exposure to four major aflatoxins (AFB_1_, AFB_2_, AFG_1_, and AFG_2_) and the associated HCC risks among residents of Guangzhou. Regarding aflatoxin contamination, AFB_1_ was the most prevalent aflatoxin, with a detection rate of 51.74%, and exhibited the highest median concentration in bulk vegetable oil (0.63 μg/kg). Although this level remains within the Chinese regulatory limit (20 μg/kg), the notably high detection rate indicates that dietary exposure to aflatoxins warrants continued attention. The predominance of AFB_1_ observed in this study is consistent with findings from Kenya and Croatia [9], where a hot and humid environment similar to that of Guangzhou favors aflatoxin production. In contrast to AFB_1_, which was detected in the largest number of samples, AFG_2_ was found less frequently but exhibited the overall highest median concentration across samples, reflecting the severity of contamination when present. This uncommon finding may be partly attributed to its relatively higher LOD, as well as a greater prevalence of AFG_2_-producing fungal strains in certain foods and potentially increased sensitivity of routine monitoring methods. However, the carcinogenic potency of AFG_2_ is considerably weaker than that of AFB_1_ based on available toxicological evidence [26]. Therefore, despite its higher median concentration, AFG_2_ should be interpreted cautiously and does not represent a greater public health concern than AFB_1_. Furthermore, this study revealed a notable co-occurrence rate of aflatoxins (52.84%), and parallel findings have been reported in other analyses: U.S. corn samples were found to contain an average of five mycotoxins per sample [30], while in Chinese instant coffee, 39.29% of samples contained two or more mycotoxins [31]. These findings underscore the complex scenario of high aflatoxin detection rates coupled with the widespread co-occurrence of multiple aflatoxins, highlighting the necessity for comprehensive surveillance strategies to assess cumulative exposure to co-occurring toxins. As the multi-aflatoxin analysis was purposefully focused on high-risk categories to enable cumulative assessment, co-occurrence patterns may not be generalizable to all monitored food categories.

Although dietary exposure to aflatoxins was low across the whole population, it varied markedly among age groups. Children aged 3–6 years exhibited the highest exposure risk (MoE: 900–1206), primarily driven by roasted peanuts—a modifiable dietary factor that could be targeted by public health interventions, such as parental guidance on snack choices. Conversely, elderly individuals showed the lowest exposure risk, possibly due to reduced consumption of nuts and vegetable oils. Unlike other age groups, where raw nuts were the primary risk food, roasted peanuts were the dominant contributor to aflatoxin exposure in the 3–6 years age group due to relatively high intake (0.3316–0.4442 ng/kg·bw). This finding aligns with previous studies that identified peanuts as a major dietary source of aflatoxins [32,33,34]. Several factors may be associated with the elevated exposure from roasted peanuts, including the high thermal stability of aflatoxins, which limits their degradation during processing [35], concentration effects due to moisture loss, and potential post-processing contamination during storage. The contrast in risk between raw/roasted peanuts and other nuts underscores the influence of processing techniques on aflatoxin levels. Moreover, these results suggest that risk management strategies should be age-specific, namely, targeted reduction in the consumption of roasted peanuts in young children and raw nuts in other populations.

In line with the age-specific exposure patterns, the estimated HCC risk attributable to dietary aflatoxin intake varied across age groups similarly, with the highest risk observed in children aged 3–6 years. In the present study, rice, rice products, and coarse grains were the primary contributors to AFB_1_-induced HCC risk for the whole population. Coarse grains dominated for individuals aged ≥60 years, whereas rice and rice products were the main contributors for younger age groups. Roasted peanuts and children aged 3–6 years represented the highest-risk food and population group for AFT-induced HCC risk. Childhood is a critical period of susceptibility due to biological factors such as immature metabolic detoxification pathways and rapid cell proliferation. In addition, children have higher food intake per kilogram of body weight, leading to greater aflatoxin exposure per unit of body weight and, consequently, elevated lifetime cancer risk. Although both the MoE approach and the HCC risk model identified similar high-risk food categories, some differences were observed. While the MoE approach emphasized foods such as flour and flour products, the HCC risk assessment highlighted foods such as coarse grains. This divergence can be explained by the models’ different considerations: MoE depends solely on EDI from food, while HCC risk additionally accounts for the HBV-susceptible population, given a strong association between dietary AFB_1_ exposure and populations with a high prevalence of chronic HBV infection [36]. The HBV-susceptible population consists primarily of older adults [12]. Thus, while a low MoE identifies foods and populations for priority control, a high HCC risk reveals critical food-consumer combinations for long-term public health interventions. This emphasizes the importance of a targeted risk assessment that analyzes exposure by age, dietary patterns, and health status. Notably, the foods identified as high HCC risk are dietary staples, and therefore, stringent quality control of these foods is essential to reduce population-wide health risks. Although the estimated HCC incidence from dietary aflatoxin exposure increase (0.015 per 100,000 person-years) appears modest at the population level, it represents a preventable burden given that dietary aflatoxin exposure is a modifiable risk factor, particularly in high-consumption subgroups.

Significant differences in aflatoxin contamination were observed between bulk and pre-packaged foods, particularly for vegetable oil and rice. Bulk vegetable oil and bulk rice exhibited significantly higher aflatoxin levels than their pre-packaged counterparts (*p* < 0.001), highlighting the influence of packaging and storage conditions on contamination. Bulk foods are often stored under less controlled conditions, with inadequate sealing and prolonged exposure to air and moisture, which can promote fungal growth and aflatoxin formation. Similar findings have been reported in a nationwide survey of vegetable oils marketed across 28 provinces in China, which identified higher aflatoxin contamination in bulk oils compared with packaged products [37]. The relatively low contamination levels in pre-packaged vegetable oil observed in this study further support previous evidence that standardized processes, improved packaging, and controlled storage conditions contribute to reduced aflatoxin contamination in pre-packaged foods [38]. These findings highlight an urgent need for technological advances in storage and packaging infrastructure. The development of sustainable active packaging materials with antimicrobial properties [39], together with the application of emerging artificial intelligence technologies to mitigate mycotoxin contamination from farm to fork [40], is anticipated to become a major focus of future research, thereby advancing economic development and social welfare.

Aflatoxin contamination also varied by retail sources. Bulk vegetable oils obtained from farmers’ markets showed significantly higher concentrations of AFB_1_ and AFB_2_ compared with those from food production workshops, while higher levels of AFG_1_ and AFG_2_ were observed in roasted nuts sold in grocery stores compared with farmers’ markets and supermarkets. Li et al. have reported comparable patterns according to which aflatoxin contamination in dried chili products sold at farmers’ markets was substantially higher than that in retail stores [41], which was attributed to differences in packaging materials and storage conditions. Products sold at farmers’ markets are often stored in materials with inferior sealing properties and displayed in open-air environments for extended periods, increasing exposure to moisture and oxygen, thus facilitating fungal growth and toxin production [42]. These findings suggest that the retail environment and postproduction handling play important roles in aflatoxin contamination. To reduce aflatoxin contamination, food producers and retailers should ensure the safety of food packaging materials, maintain strict storage conditions, and prevent ambient exposure. Moreover, targeted inspections and mandatory labeling of production dates and origins at farmers’ markets can protect disadvantaged consumers dependent on these inexpensive food sources, facilitating fair resource distribution and advancing sustainable development.

The strengths of this study include prolonged surveillance, systematic coverage of packaging types and retail source diversity, and dual-model risk assessment (MoE and HCC), providing age-stratified insights into multi-aflatoxin exposure and associated health risks. This study is designed as a dietary exposure and risk assessment for four aflatoxins in the population. Accordingly, the results are intended to estimate population-level risk and should not be interpreted as evidence of causality between aflatoxin exposure and liver cancer. These findings underscore the need for targeted risk management strategies focusing on high-risk foods, vulnerable population groups, and critical points along the food supply chain. Improved packaging types and storage conditions may help reduce dietary aflatoxin exposure and associated health risks. This study has direct implications for food safety regulation in Guangzhou and other regions with a hot, humid climate conducive to mycotoxin proliferation. Beyond Guangzhou, the analytical framework and risk assessment approach employed here, particularly the age-specific exposure assessment, are extendable to regions facing similar aflatoxin burdens, such as Southeast Asia, Sub-Saharan Africa, and the southern United States. However, several limitations should be acknowledged. Only a subset of food categories was simultaneously analyzed for all four aflatoxins, and up-to-date food consumption data for Guangzhou residents were not available. Despite the generally stable dietary pattern in Guangzhou, relying on 2011 consumption data may still introduce a certain degree of exposure misclassification bias, for instance, due to temporary changes during events such as the COVID-19 pandemic. Additionally, HCC risk calculations did not account for confounding factors such as alcohol consumption and metabolic diseases due to data limitations, which may influence the estimation of the attributable HCC risk. Future studies should consider more comprehensive food coverage, combined exposure to multiple mycotoxins, and the incorporation of biomonitoring data to better characterize internal exposure. Temperature and atmospheric CO_2_ levels significantly affect mycotoxigenic fungal contamination in crops [43], and aflatoxin contamination can exhibit seasonality. Future risk assessments should incorporate climate and temporal variables alongside up-to-date food consumption data across Guangzhou’s diverse districts to comprehensively evaluate population health risks.

## 5. Conclusions

This study evaluated age-specific cumulative dietary exposure to co-occurring aflatoxins and HCC risk in Guangzhou, China. The results indicated notable aflatoxin contamination rates and frequent co-occurrence of multiple aflatoxins in food samples. For the general population, most foods posed a low dietary exposure risk. However, roasted peanuts and raw nuts exhibited higher exposure risks, especially for children aged 3–6 years. Rice and rice products were the primary contributors to the estimated HCC risk for most age groups, whereas coarse grains accounted for the highest risk among older adults under the current cumulative exposure assessment. Aflatoxin contamination was also more pronounced in bulk products and varied by retail source. By integrating age-stratified dietary consumption data with multi-toxin contamination and cumulative exposure assessment, this replicable framework can help identify vulnerable age groups in other high-risk regions and provide preliminary evidence to support targeted food safety regulation and age-specific dietary guidance in Guangzhou and similar hot and humid regions.

## Figures and Tables

**Table 1:** The specified maximum limits for AFB_1_ and AFT in foods.

Aflatoxin	Food ^a^	Maximum Limit (μg/kg)	Reference Standard
AFB_1_	Rice and rice products	10	GB 2761-2017 [16]
	Flour and flour products	5	
	Coarse grains	20	
	Peanuts	20	
	Other nuts	5	
	Vegetable oil	20	
AFT ^b^	Peanuts	15	Codex Standard 193-1995 [20]

^a^ Maximum limited levels of aflatoxins in edible portions of raw and/or finished foods; ^b^ AFT refers to the sum of AFB_1_, AFB_2_, AFG_1_, and AFG_2_.

**Table 2:** Age-stratified food consumption in Guangzhou.

Food Group	Age Group									
	3–6 Years		7–17 Years		18–59 Years		≥60 Years		Whole Population	
	Mean ^a^	SD ^b^	Mean	SD	Mean	SD	Mean	SD	Mean	SD
Rice and rice products	78.56	45.48	121.26	73.83	146.47	90.46	126.54	90.71	135.84	86.94
Flour and flour products	32.58	22.32	48.42	38.18	52.33	38.69	50.62	33.10	49.33	36.29
Coarse grains	4.00	17.98	5.11	16.05	7.14	24.56	9.85	41.01	6.57	23.77
Other nuts ^c^	2.43	14.46	2.22	7.57	2.41	12.00	0.57	2.70	2.30	11.22
Starchy foods	0.76	6.88	1.57	10.66	1.49	12.05	0.55	5.58	1.39	11.13
Peanuts	1.47	10.22	0.51	3.52	0.65	6.98	0.06	0.50	0.66	6.63
Vegetable oil	11.33	9.62	22.34	18.50	26.41	17.28	9.57	5.33	26.62	16.78

^a^ Mean daily consumption (g/day) of various foods among five age groups in Guangzhou; ^b^ SD: standard deviation; ^c^ other nuts include walnuts, sunflower seeds, and similar products.

## Data Availability

The detailed raw data used in this study are derived from government monitoring programs and cannot be made publicly available due to confidentiality requirements. All data supporting the findings are provided in the Section 3 and Supplementary Tables in this article. Further inquiries can be directed to the corresponding author.

## Supplementary Materials

The following supporting information can be downloaded at: https://www.mdpi.com/article/10.3390/foods15111839/s1, Table S1: Detection rates and median concentrations of aflatoxins in different food groups; Table S2: Aflatoxin concentrations in foods by different packaging types; Table S3: Aflatoxin concentrations in foods collected from different retail sources; Table S4: Dietary exposure of AFT by different age groups; Table S5: Risk characterization of AFT exposure by different age groups based on the MoE approach; Table S6: Risk characterization of AFB_1_ exposure by different age groups based on the MoE approach; Table S7: Hepatocellular carcinoma (HCC) risk (cases/100,000 population) of AFB1 among different age groups; Table S8: Hepatocellular carcinoma (HCC) risk (cases/100,000 population) of AFT among different age groups.

## Abbreviations

The following abbreviations are used in this manuscript:

AFB_1_	Aflatoxin B_1_
AFB_2_	Aflatoxin B_2_
AFG_1_	Aflatoxin G_1_
AFG_2_	Aflatoxin G_2_
AFT	Total Aflatoxins
BMDL_10_	Benchmark Dose Lower Confidence Limit for a 10% Response
EDI	Estimated Daily Intake
EFSA	European Food Safety Authority
HBV	Hepatitis B Virus
HCC	Hepatocellular Carcinoma
LB	Lower Bound
LOD	Limit of Detection
MB	Middle Bound
MoE	Margin of Exposure
ND	Non-Detects
SD	Standard Deviation
UB	Upper Bound
WHO	World Health Organization
