# Supplementary material for: Multiple Aflatoxins Drive Cumulative Dietary Exposure and Hepatocellular Carcinoma Risk: An Age-Stratified Study in Guangzhou, China
Source: Foods. 2026 May 22;15(11):1839. doi: 10.3390/foods15111839 (PMC13257253; doi:10.3390/foods15111839)
Supplement: Supplementary file 1 [file foods-15-01839-s001.zip › foods-4298083-supplementary.pdf]

Supplementary Material

Multiple Aflatoxins Drive Cumulative Dietary Exposure and Hepatocellular Carcinoma Risk: An Age-Stratified Study in Guangzhou, China

Qian Huang, Yanyan Wang, Yan Li, Yixuan Xu, Yuhua Zhang, Lan Liu, Jinheng Zeng, Weiwei Zhang, Yan Yang

Supplementary Table S1. Detection rates and median concentrations of aflatoxins in different food groups.

Food group	*N* [a]	AFB_1					AFB_2				AFG_1				AFG_2				AFT [g]			
		n(%) [b]	n_{ex}(%) [c]	Median (ug/kg)			*n*(%)	Median (ug/kg)			*n*(%)	Median (ug/kg)			*n*(%)	Median (ug/kg)			*n*(%)	Median (ug/kg)		
				LB [d]	MB [e]	UB [f]		LB	MB	UB		LB	MB	UB		LB	MB	UB		LB	MB	UB
**Bulk vegetable oil**	697	471(67.58)	50（7.17）	0.63	0.63	0.63	275(39.45)	0.00	0.13	0.25	9(1.29)	0.00	0.50	1.00	27(3.87)	0.00	0.50	1.00	479(68.72)	0.73	1.76	2.78
**Pre-packaged vegetable oil**	40	18(45.00)	0（0.00）	0.00	0.13	0.25	5(12.50)	0.00	0.13	0.25	0(0.00)	0.00	0.13	0.25	6(15.00)	0.00	0.13	0.25	18(45.00)	0.00	0.50	1.00
**Roasted peanuts**	139	44(31.65)	2（1.44）	0.00	0.13	0.25	13(9.35)	0.00	0.13	0.25	0(0.00)	0.00	0.25	0.50	0(0.00)	0.00	0.50	1.00	45(32.37)	0.00	1.00	2.00
**Other roasted nuts**	207	41(19.81)	1（0.48）	0.00	0.13	0.25	3(1.45)	0.00	0.13	0.25	0(0.00)	0.00	0.25	0.50	2(0.97)	0.00	0.50	1.00	45(21.74)	0.00	1.00	2.00
**Raw peanuts**	46	6(13.04)	0（0.00）	0.00	0.13	0.25	3(6.52)	0.00	0.13	0.25	0(0.00)	0.00	0.25	0.50	0(0.00)	0.00	0.50	1.00	6(13.04)	0.00	1.00	2.00
**Other raw nuts**	50	30(60.00)	1（2.00）	0.45	0.45	0.45	1(2.00)	0.00	0.13	0.25	1(2.00)	0.00	0.25	0.50	1(2.00)	0.00	0.50	1.00	30(60.00)	0.45	1.33	2.20
**Total**	1179	610(51.74)	54（4.58）	0.29	0.29	0.29	300(25.45)	0.00	0.13	0.25	10(0.85)	0.00	0.25	0.50	36(3.05)	0.00	0.50	1.00	623(52.84)	0.32	1.25	2.50

[a] ***N***: total numbersof samples in different food group; n: number of samples with detectable levels of aflatoxins (>LOD)

[b] ***n***(%): number and percentage of samples with detectable levels of aflatoxins

[c] n_{ex}(%): number and percentage of samples exceeding the limit GB 2761-2017 or Codex Standard 193-1995

[d] **LB**: lower bound, if aflatoxin concentration value < LOD, value=0;

[e] **MB**: middle bound, value= ½ LOD

[f] **UB**: upper bound, value=LOD

[g] **AFT**:$\sum AFB_1+AFB_2+AFG_1+AFG_2.$

Supplementary Table S2. Aflatoxin concentrations in foods by different packaging types.

Food group	Aflatoxin	Packaging type				*Z*	*P* [b]
		Bulk		Pre-packaged			
		Median[a] (ug/kg)	(P_{25},P_{75}) [a]	Median (ug/kg)	(P_{25},P_{75})		
**Vegetable oil**	**AFB_1**	1.40	(0.64,6.14)	0.13	(0.13,0.67)	-6.683	<0.001
	**AFB_2**	0.65	(0.13,1.50)	0.13	(0.13,0.13)	-3.682	<0.001
	**AFG_1**	0.25	(0.13,0.25)	0.13	(0.13,0.13)	-10.470	<0.001
	**AFG_2**	0.50	(0.33,0.50)	0.13	(0.13,0.68)	-6.271	<0.001
	**AFT**	3.31	(1.51,7.86)	0.50	(0.13,1.23)	-13.148	<0.001
**Nuts**	**AFB_1**	0.13	(0.13,0.13)	0.13	(0.13,0.13)	-1.341	0.180
	**AFB_2**	0.13	(0.13,0.13)	0.13	(0.13,0.13)	-1.437	0.151
	**AFG_1**	0.13	(0.13,0.13)	0.13	(0.13,0.13)	-0.987	0.324
	**AFG_2**	0.13	(0.13,0.13)	0.13	(0.13,0.13)	-1.027	0.304
	**AFT**	0.50	(0.50,1.00)	1.00	(0.50,1.00)	-1.424	0.155
**Rice**	**AFB_1**	0.13	(0.13,0.13)	0.13	(0.13,0.13)	-2.932	0.003
**Flour**	**AFB_1**	0.13	(0.13,0.13)	0.13	(0.13,0.13)	-0.018	0.985

[a] The calculation of the Median, P_{25} and P_{75} is based on the middle bound concentration

[b] *P*: A Mann–Whitney test was used for analyzing aflatoxin concentrations in foods by different packaging types

Supplementary Table S3. Aflatoxin concentrations in foods collected from different retail sources.

Food group	Aflatoxin	Retail source	Parameter				
			Median [a] (μg/kg)	(P_{25},P_{75}) [a]	*H*	*P* [b]	P_{post} [c]
Bulk vagetable oil	AFB_1	Eateries	0.13	(0.13–2.27)	11.98	0.007	
		Farmers' markets	2.12	(0.31–5.68)			0.012 (Farmers' markets vs Food production workshops)
		Food production workshops	0.57	(0.13–2.98)			
		Online stores	0.71	(0.33–4.54)			
	AFB_2	Eateries	0.13	(0.13–1.45)	10.29	0.016	
		Farmers' markets	0.31	(0.13–0.91)			0.010 (Farmers' markets vs Food production workshops)
		Food production workshops	0.13	(0.13–0.50)			
		Online stores	0.13	(0.13–0.56)			
	AFG_1	Eateries	0.25	(0.25–0.25)	3.78	0.151	
		Farmers' markets	0.50	(0.25–0.50)			
		Food production workshops	0.50	(0.25–0.50)			
		Online stores	0.50	(0.25–0.50)			
	AFG_2	Eateries	0.50	(0.50–0.50)	1.49	0.475	
		Farmers' markets	0.50	(0.50–0.50)			
		Food production workshops	0.50	(0.50–0.50)			
		Online stores	0.50	(0.50–0.50)			
	AFT[d]	Eateries	1.00	(1.00–4.47)	15.75	0.001	
		Farmers' markets	3.49	(1.44–7.45)			0.002 (Farmers' markets vs Food production workshops)
		Food production workshops	1.64	(1.25–4.72)			
		Online stores	1.83	(1.30–6.00)			
Pre-packed vegetable oil	AFB_1	Farmers' markets	0.13	(0.13–1.09)	0.10	0.751	
		Supermarkets	0.13	(0.13–0.96)			
	AFB_2	Farmers' markets	0.13	(0.13–0.16)			
		Supermarkets	0.13	(0.13–0.13)			
	AFG_1	Farmers' markets	0.13	(0.13–0.13)	0.18	0.671	
		Supermarkets	0.13	(0.13–0.13)			
	AFG_2	Farmers' markets	0.13	(0.13–0.48)			
		Supermarkets	0.13	(0.13–0.72)			
	AFT	Farmers' markets	0.50	(0.50–1.99)	0.03	0.856	
		Supermarkets	0.50	(0.50–1.87)			
Roasted peanuts	AFB_1	Farmers' markets	0.13	(0.13–0.22)	1.83	0.608	
		Grocery stores	0.13	(0.13–0.54)			
		Supermarkets	0.13	(0.13–0.47)			
		Online stores	0.13	(0.13–0.75)			
	AFB_2	Farmers' markets	0.13	(0.13–0.13)	0.43	0.806	

		Grocery stores	0.13	(0.13–0.13)			
		Supermarkets	0.13	(0.13–0.13)			
		Online stores	0.13	(0.13–0.13)			
	**AFG_1**	Farmers' markets	0.25	(0.13–0.25)	3.53	0.171	
		Grocery stores	0.25	(0.19–0.25)			
		Supermarkets	0.25	(0.13–0.25)			
		Online stores	0.25	(0.25–0.25)			
	**AFG_2**	Farmers' markets	0.50	(0.13–0.50)	3.07	0.215	
		Grocery stores	0.50	(0.31–0.50)			
		Supermarkets	0.50	(0.13–0.50)			
		Online stores	0.50	(0.50–0.50)			
	**AFT**	Farmers' markets	1.00	(0.50–1.00)	7.51	0.057	0.043 (Farmers' markets vs Online stores)
		Grocery stores	1.00	(1.00–1.41)			
		Supermarkets	1.00	(0.71–1.37)			
		Online stores	1.00	(1.00–1.62)			
**Raw peanuts**	**AFB_1**	Farmers' markets	0.13	(0.13–0.13)	0.61	0.894	
		Grocery stores	0.13	(0.13–0.13)			
		Supermarkets	0.13	(0.13–0.13)			
		Online stores	0.13	(0.13–0.13)			
	**AFB_2**	Farmers' markets	0.13	(0.13–0.13)	0.01	0.915	
		Grocery stores	0.13	(0.13–0.13)			
		Supermarkets	0.13	(0.13–0.13)			
		Online stores	0.13	(0.13–0.13)			
	**AFG_1**	Farmers' markets	0.25	(0.25–0.25)	0.00	1.000	
		Grocery stores	0.25	(0.25–0.25)			
		Supermarkets	0.25	(0.25–0.25)			
		Online stores	0.25	(0.25–0.25)			
	**AFG_2**	Farmers' markets	0.50	(0.50–0.50)	0.00	1.000	
		Grocery stores	0.50	(0.50–0.50)			
		Supermarkets	0.50	(0.50–0.50)			
		Online stores	0.50	(0.50–0.50)			
	**AFT**	Farmers' markets	1.00	(1.00–1.00)	0.61	0.894	
		Grocery stores	1.00	(1.00–1.00)			
		Supermarkets	1.00	(1.00–1.00)			
		Online stores	1.00	(1.00–1.00)			
**Other roasted nuts**	**AFB_1**	Farmers' markets	0.13	(0.13–0.13)	1.46	0.692	
		Grocery stores	0.13	(0.13–0.13)			
		Supermarkets	0.13	(0.13–0.13)			
		Online stores	0.13	(0.13–0.13)			
	**AFB_2**	Farmers' markets	0.13	(0.13–0.13)	0.34	0.557	
		Grocery stores	0.13	(0.13–0.13)			
		Supermarkets	0.13	(0.13–0.13)			
		Online stores	0.13	(0.13–0.13)			
	**AFG_1**	Farmers' markets	0.13	(0.13–0.25)	10.38	0.006	0.027 (vs Grocery stores)

		Grocery stores	0.25	(0.13–0.25)			0.007 (vs Supermarkets)
		Supermarkets	0.13	(0.13–0.25)			
		Online stores	0.25	(0.25–0.25)			
	**AFG_2**	Farmers' markets	0.13	(0.13–0.50)	11.51	0.003	0.014 (vs Grocery stores)
		Grocery stores	0.50	(0.13–0.50)			0.005 (vs Supermarkets)
		Supermarkets	0.13	(0.13–0.50)			
		Online stores	0.50	(0.50–0.50)			
	**AFT**	Farmers' markets	0.50	(0.50–1.00)	26.60	0.000	0.048 (vs Grocery stores)<br>0.000 (vs Online stores)
		Grocery stores	1.00	(0.50–1.00)			0.018 (vs Supermarkets)<br>0.031 (vs Online stores)
		Supermarkets	0.50	(0.50–1.00)			0.000 (vs Online stores)
		Online stores	1.00	(1.00–1.00)			
**Other raw nuts**	**AFB1**	Farmers' markets	0.52	(0.13–0.66)	1.53	0.676	
		Grocery stores	0.39	(0.13–0.71)			
		Supermarkets	0.48	(0.45–0.53)			
		Online stores	0.13	(0.13–0.58)			
	**AFB_2**	Farmers' markets	0.13	(0.13–0.13)	0.00	1.000	
		Grocery stores	0.13	(0.13–0.13)			
		Supermarkets	0.13	(0.13–0.13)			
		Online stores	0.13	(0.13–0.13)			
	**AFG_1**	Farmers' markets	0.25	(0.25–0.25)	0.00	1.000	
		Grocery stores	0.25	(0.25–0.25)			
		Supermarkets	0.25	(0.25–0.25)			
		Online stores	0.25	(0.25–0.25)			
	**AFG_2**	Farmers' markets	0.50	(0.50–0.50)	0.00	1.000	
		Grocery stores	0.50	(0.50–0.50)			
		Supermarkets	0.50	(0.50–0.50)			
		Online stores	0.50	(0.50–0.50)			
	**AFT**	Farmers' markets	1.40	(1.00–1.53)	1.67	0.644	
		Grocery stores	1.27	(1.00–1.60)			
		Supermarkets	1.36	(1.32–1.40)			
		Online stores	1.00	(1.00–1.46)			

[a] Median, P_{25}, P_{75} were calculated based on the middle bound concentration

[b] *P*: Aflatoxins levels differed among all retail sources in one food group using a Kruskal–Wallis test

[c] P_{post}: Pairwise differences in aflatoxin levels between retail sources using a Dunn test, only p values < 0.05 are shown

[d] AFT: $\Sigma AFB_1+AFB_2+AFG_1+AFG_2$.

Supplementary Table S4. Dietary exposure of AFT by different age groups.

Food group	EDI [a]（ng/kg·bw）														
	3-6 years			7-17 years			18-59 years			≥60 years			whole population		
	LB [b]	MB [c]	UB [d]	LB	MB	UB	LB	MB	UB	LB	MB	UB	LB	MB	UB
**Bulk vegetable oil**	0.0478	0.0545	0.0613	0.0032	0.0037	0.0042	0.0242	0.0275	0.0310	0.0000	0.0000	0.0000	0.0183	0.0209	0.0234
**Pre-packed vegetable oil**	0.0074	0.0100	0.0126	0.0004	0.0006	0.0008	0.0038	0.0051	0.0064	0.0000	0.0000	0.0000	0.0028	0.0037	0.0174
**Roasted peanuts**	0.3316	0.3879	0.4442	0.0578	0.0675	0.0775	0.0472	0.0552	0.0633	0.0043	0.0050	0.0057	0.0500	0.0586	0.0671
**Other roasted nuts**	0.0213	0.1113	0.2013	0.0098	0.0508	0.0919	0.0069	0.0357	0.0644	0.0016	0.0087	0.0157	0.0068	0.0352	0.0635
**Raw peanuts**	0.0324	0.1040	0.1755	0.0056	0.0181	0.0306	0.0046	0.0148	0.0249	0.0005	0.0014	0.0023	0.0049	0.0157	0.0265
**Other raw nuts**	0.0822	0.1926	0.3029	0.0377	0.0880	0.1384	0.0263	0.0617	0.0968	0.0063	0.0150	0.0236	0.0259	0.0608	0.0956

[a] EDI: estimated daily intake

[b] LB: lower bound, if aflatoxin concentration value < LOD, value=0

[c] MB: middle bound, value= ½ LOD

[d] UB: upper bound, value=LOD

Supplementary Table S5. Risk characterization of AFT exposure by different age groups based on the MoE approach.

Food group	MoE$_{total}$ [a]														
	3-6 years			7-17 years			18-59 years			≥60 years			whole population		
	LB [b]	MB [c]	UB [d]	LB	MB	UB	LB	MB	UB	LB	MB	UB	LB	MB	UB
**Bulk vegetable oil**	8375	7344	6525	124706	108108	95238	16517	14545	12903	/	/	/	21872	19139	17094
**Pre-packed vegetable oil**	53709	40000	31746	943953	666667	500000	105938	78431	62500	/	/	/	141176	108108	22989
**Roasted peanuts**	1206	1031	900	6920	5926	5161	8472	7246	6319	92770	80000	70175	8002	6826	5961
**Other roasted nuts**	18742	3594	1987	40770	7874	4353	58158	11204	6211	247076	45977	25478	59026	11364	6299
**Raw peanuts**	12344	3846	2279	71233	22099	13072	87253	27027	16064	882613	285714	173913	81305	25478	15094
**Other raw nuts**	4867	2077	1321	10617	4545	2890	15212	6483	4132	63314	26667	16949	15455	6579	4184

[a] MoE$_{total}$: the total margin of exposure was derived from the individual MoE values of AFB_1, AFB_2, AFG_1, and AFG_2 based on formula (3) described in the article. MoE<10,000 means a potential public health concern

[b] LB: lower bound, if aflatoxin concentration value < LOD, value=0

[c] MB: middle bound, value= ½ LOD

[d] UB: upper bound, value=LOD

Supplementary Table S6. Risk characterization of AFB_1 exposure by different age groups based on the MoE approach.

Food groups	MoE [a]														
	3-6 years			7-17 years			18-59 years			≥60 years			whole population		
	LB [b]	MB [c]	UB [d]	LB	MB	UB	LB	MB	UB	LB	MB	UB	LB	MB	UB
Rice and rice products	/	815	407	/	1056	528	/	1355	677	/	1283	642	/	1413	707
Flour and flour products	/	1964	982	/	2644	1322	/	3791	1896	/	3581	1790	/	3845	1923
Starchy foods	/	84433	42216	/	81477	40738	/	133065	66533	/	347826	173913	/	138528	69264
Coarse grains	6346	6346	6346	12692	9946	9946	11028	11028	11028	7737	7737	7737	11590	11590	11590
Bulk vegetable oil	85901	85901	85901	171803	1256874	1256874	169793	169793	169793	/	/	/	223881	223881	223881
Pre-packed vegetable oil	/	460432	230216	/	6736842	3368421	/	910092	455046	/	/	/	/	1200000	600000
Roasted peanuts	/	43656	21828	/	250489	125245	/	306646	153323	/	3368421	1684211	/	289157	144578
Other roasted nuts	/	26337	13169	/	57632	28816	/	82290	41145	/	338028	169014	/	83478	41739
Raw peanuts	/	43656	21828	/	250489	125245	/	306646	153323	/	3368421	1684211	/	289157	144578
Other raw nuts	7316	7316	7316	16009	16009	16009	22858	22858	22858	93897	93897	93897	23188	23188	23188

[a] MoE: the margin of exposure was calculated based on formula (2) described in the article. MoE<10,000 means a potential public health concern.

[b] LB: lower bound, if aflatoxin concentration value < LOD, value=0

[c] MB: middle bound, value= ½ LOD

[d] UB: upper bound, value=LOD.

Supplementary Table S7. Hepatocellular carcinoma (HCC) risk (cases/100,000 population) of AFB_1 among different age groups.

Food group [a]	HCC individual	HCC risk [b]				
		3-6 years	7-17 years	18-59 years	≥60 years	whole population
Rice and rice products	P_{HBsAg+} [c]	0.1579	0.1219	0.0950	0.1003	0.0910
	P_{HBsAg-} [d]	0.0053	0.0041	0.0032	0.0033	0.0030
	$P_{Combined}$ [e]	0.0146	0.0112	0.0088	0.0093	0.0084
Flour and flour products	P_{HBsAg+}	0.0645	0.0479	0.0334	0.0354	0.0330
	P_{HBsAg-}	0.0022	0.0016	0.0011	0.0012	0.0011
	$P_{Combined}$	0.0060	0.0044	0.0031	0.0033	0.0030
Coarse grains	P_{HBsAg+}	0.1512	0.0965	0.0870	0.1240	0.0828
	P_{HBsAg-}	0.0050	0.0032	0.0029	0.0041	0.0028
	$P_{Combined}$	0.0140	0.0089	0.0080	0.0114	0.0076
Roasted peanuts	P_{HBsAg+}	0.0778	0.0136	0.0111	0.0010	0.0118
	P_{HBsAg-}	0.0104	0.0018	0.0015	0.0001	0.0016
	$P_{Combined}$	0.0072	0.0013	0.0010	0.0001	0.0011

[a] Based on the MoE results for AFB_1 (Supplementary Table 6), food groups presenting relatively higher exposure risks were selected for HCC risk assessment

[b] The calculation of the HCC risk is based on the middle bound value of EDI of AFB_1

[c] P_{HBsAg+}: HCC risk for $HBsAg^+$ individuals exposed to AFB1 in the population

[d] P_{HBsAg-}: HCC risk for HBsAg– individuals exposed to AFB_1 in the population

[e] Pcombined: Total HCC risk for the population exposed to AFB_1.

Supplementary Table S8. Hepatocellular carcinoma (HCC) risk (cases/100,000 population) of AFT among different age groups.

Food group [a]	HCC individual	HCC risk [b]				
		3-6 years	7-17 years	18-59 years	≥60 years	whole population
Roasted peanuts	P_{HBsAg+} [c]	0.1164	0.0203	0.0166	0.0015	0.0176
	P_{HBsAg-} [d]	0.0039	0.0007	0.0006	0.0001	0.0006
	$P_{Combined}$ [e]	0.0146	0.0025	0.0021	0.0002	0.0022
Other raw nuts	P_{HBsAg+}	0.0578	0.0264	0.0185	0.0045	0.0182
	P_{HBsAg-}	0.0019	0.0009	0.0006	0.0002	0.0006
	$P_{Combined}$	0.0072	0.0033	0.0023	0.0006	0.0023
Other roasted nuts	P_{HBsAg+}	0.0334	0.0152	0.0107	0.0026	0.0106
	P_{HBsAg-}	0.0011	0.0005	0.0004	0.0001	0.0004
	$P_{Combined}$	0.0042	0.0019	0.0013	0.0003	0.0013

[a] Based on the MoE results for AFT (Supplementary Table 5), food groups presenting relatively higher exposure risks were selected for HCC risk assessment

[b] The calculation of the HCC risk is based on the middle bound value of EDI of AFT

[c] P_{HBsAg+}: HCC risk for $HBsAg^+$ individuals exposed to AFT in the population

[d] P_{HBsAg-}: HCC risk for HBsAg–individuals exposed to AFT in the population

[e] Pcombined: Total HCC risk for the population exposed to AFT
